# Significance of searching for ganglia in the terminal rectum/fistula of complex anorectal malformations: Related to defecation function

**DOI:** 10.3389/fped.2023.1124647

**Published:** 2023-02-27

**Authors:** Wei Feng, Huaqi Mu, Minmin Chen, Jin Zhu, Chenzhu Xiang, Linxiao Fan, Jinping Hou, Xiaohong Die, Yi Wang

**Affiliations:** ^1^ Department of General and Neonatal Surgery, Children's Hospital of Chongqing Medical University, National Clinical Research Center for Child Health and Disorders, Ministry of Education Key Laboratory of Child Development and Disorders, Chongqing Key Laboratory of Pediatrics, Chongqing, China; ^2^ Department of Pathology, Children's Hospital of Chongqing Medical University, Chongqing, China

**Keywords:** anorectal malformation, ganglia, defecation function, constipation, enteric nervous system

## Abstract

**Background:**

The need to search for ganglia in the terminal rectum/fistula of complex anorectal malformations (ARMs) remains controversial. This study aims to evaluate the relationship between ganglia absence in the terminal rectum/fistula and defecation function after anoplasty.

**Methods:**

A retrospective review of patients who received anoplasty for treating male imperforate anus with rectobulbar (RB)/rectoprostatic (RP) fistulas at a tertiary pediatric hospital was conducted with registered demographic data, imaging study results, and information on the terminal rectum/fistula specimen (excision extension and pathological findings). According to the pathological findings, patients were divided into Groups 1 (ganglia absence) and 2 (ganglia presence). Furthermore, the postoperative defecation function was evaluated using various rating scale questionnaires. Statistical analysis was performed using SPSS 22.0.

**Results:**

Of the 62 patients, 18 (29.0%) showed ganglia absence in the terminal rectum/fistula. By analyzing the imaging data, spinal anomalies and spinal cord anomalies were found in 30.6% (19/62) and 56.5% (35/62) of patients, respectively. Baseline information was comparable between Groups 1 and 2 (*P* > 0.05). For defecation function, there were no significant differences in Kelly scores between the two groups (4.0 ± 0.8 vs. 4.4 ± 1.1, *P* = 0.177), while Krickenbeck (3.7 ± 1.8 vs. 5.2 ± 1.4) and Rintala (13.7 ± 3.6 vs. 16.0 ± 2.7) scores in Group 1 were significantly lower than those in Group 2 (both *P* < 0.05). The overall incidence of constipation was 50% (31/62), being higher for Group 1 than Group 2 (77.5% vs. 38.6%, *P* = 0.002). The area under the curve of ganglia absence for predicting constipation was 0.696, with 77.8% sensitivity and 61.4% specificity.

**Conclusion:**

Ganglia absence in the terminal rectum/fistula of male imperforate anus with RB/RP fistulas is associated with constipation after anoplasty, but it has limited predictive value for postoperative constipation. It is necessary to search for ganglia in the terminal rectum/fistula, both intraoperatively and postoperatively.

## Introduction

Anorectal malformation (ARM) is a common congenital malformation of the hindgut, in which the anus fails to open normally onto the perineum (1, 2). Anus reconstruction for complex ARMs, including imperforate anus without a fistula, rectobulbar (RB) fistula, rectoprostatic (RP) fistula, rectobladderneck fistula, and cloacal malformations, is a technical challenge for pediatric surgeons (3, 4). However, it is worth noting that even after anatomical reconstruction of the anus, most children still face varying degrees of functional defecation disorder (FDD), including fecal incontinence, soiling, and constipation (5, 6). In recent years, with the improvement of surgical techniques and perioperative management programs, the incidence of postoperative fecal incontinence has decreased but that of constipation has increased, which seriously affects the quality of life of patients (7–9).

The relationship between ganglionic distribution in the terminal rectum/fistula of ARMs and postoperative defecation function remains controversial. Studies have reported that neuronal dysplasia or absence might contribute to the high incidence of postoperative constipation (10, 11). Gangopadhyay et al. performed a detailed histological study of the terminal rectum/fistula and suggested excising this region for the reconstruction of ARMs (12), as did Lombardi et al. (13). However, a study conducted by Holschneider et al. showed that partial denervation of the rectum might not be the only cause of postoperative constipation and recommended using the distal rectal pouch and parts of the fistula in the reconstruction (14). Even more surprising, Uemura et al. found that ganglionic distribution was similar in the terminal rectum/fistula of ARMs and in a normal anal canal (15). By reviewing the relevant literature, we found that the above controversial findings may be due to the pathological specimens derived from different pathological types of ARMs. As we know, those with low malformations (rectoperineal and rectovestibular fistulas) usually have considerable postoperative defecation function, and the excision in such patients is less radical.

By optimizing the study design, this study aims to evaluate the relationship between ganglia absence in the terminal rectum/fistula of male imperforate anus with RB/RP fistulas and defecation function after PSARP. We hypothesized that the absence of ganglia would increase the risk of postoperative constipation.

## Materials and methods

### Study approval

This study was approved by the Institutional Research Ethics Board of the Children's Hospital affiliated with Chongqing Medical University (Date: 2021/No: 391) and complies with the 1964 Helsinki declaration and its later amendments or comparable ethical standards. Because this was a retrospective study, the requirement for informed consent was waived.

### Study population

**Figure 1:**
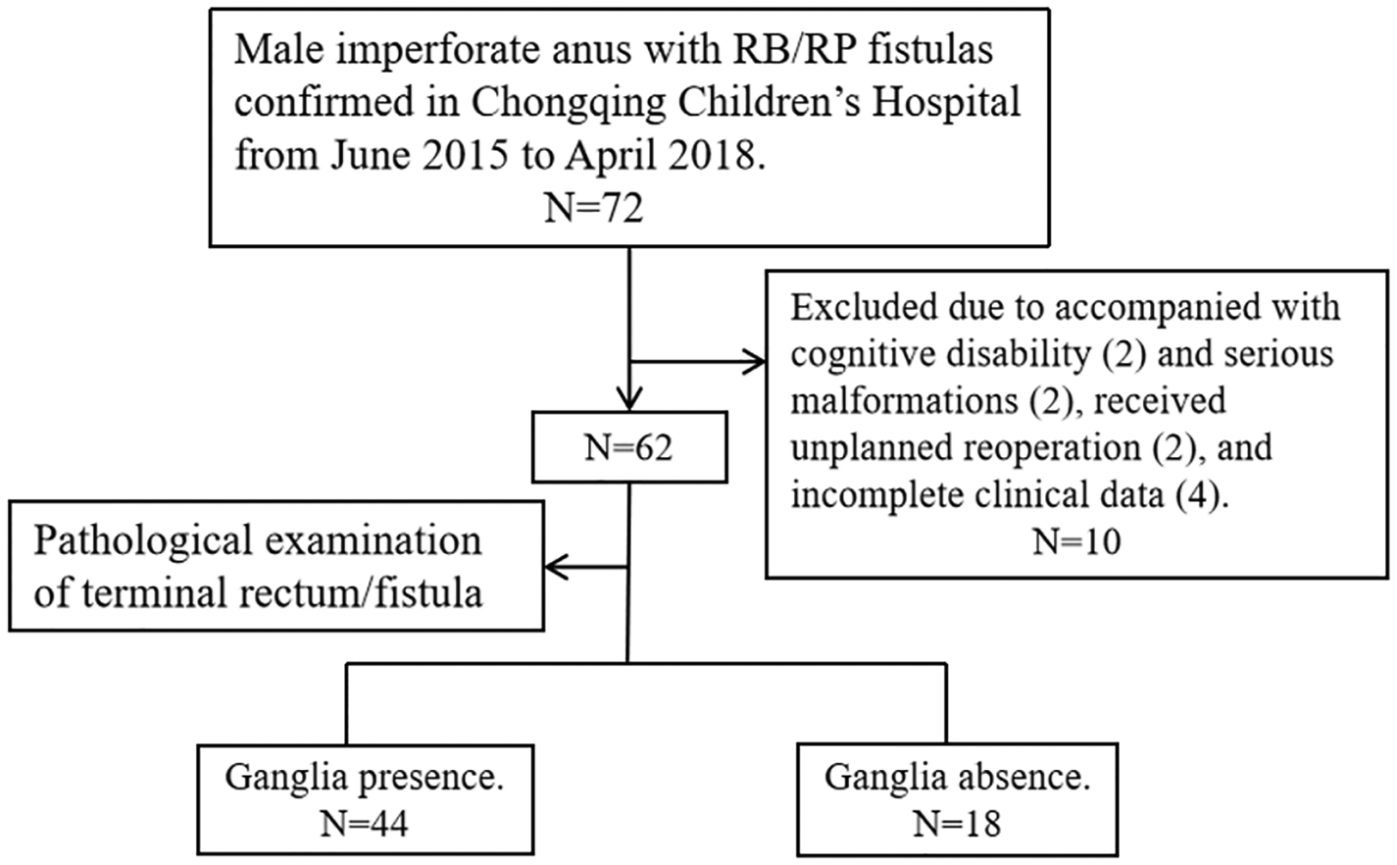
Flow diagram of the study population.

We reviewed the files of ARM patients who received PSARP for treating male imperforate anus with RB/RP fistulas in the Gastrointestinal Neonatal Surgery Department of the Children's Hospital affiliated with Chongqing Medical University (a tertiary pediatric hospital and National Clinical Research Center for Child Health and Disorders in China) during the period from June 2015 to April 2018. Furthermore, only patients who had complete clinical data and who had cooperated with the follow-up process were included in the final data set. Patients were excluded if cognitive disabilities that influence the evaluation of defecation function were identified (two cases); they received unplanned reoperation due to complications, like rectal prolapse (one case) and secondary megarectum (one case); they had serious malformations that affect the management process of ARM (i.e., solitary kidney with renal failure, hermaphroditism); or they missed information in one or more variables (four cases). Figure 1 shows a flow diagram for the inclusion and exclusion of patients in this study.

In our hospital, the patients with RB/RP fistulas underwent three-stage repair, as described below. In stage one, transverse-loop colostomy was usually performed within 48 h of birth. In stage two, PSARP, as described by Peña et al. (4), was performed 3–6 months after colostomy by the permanent team (Yi Wang and Jinping Hou, two specialist pediatric surgeons who have extensive experience and received systematic training at Cincinnati Children's Hospital Medical Center). For PSARP, distal pressure colostography (water-soluble contrast medium), performed to identify the location of the fistula, is mandatory for definitive preoperative surgical planning. Afterward, the transanal tubes were usually placed *in situ* for 7 days, and anal dilatation began in the second week and lasted for 3–6 months. The Hegar size of the anal dilator gradually increased from 8 to 12, even 14. In stage three, the colostomy was closed 3 months after PSARP. All patients started toilet training at around the age of 2 years.

### Study design

Clinical data were collected from the patient's medical records, including birth weight, gestational age, type of malformation (RB or RP fistula), living condition (urban or rural), excision length of the terminal rectum/fistula, pathological findings of terminal rectum/fistula, and imaging study results (spinal MRI and x-ray). Furthermore, we recorded four time points: age at the time of colostomy (Age 1), PSARP (Age 2), colostomy closure (Age 3), and evaluation of defecation function (Age 4).

For each patient with RB/RP fistulas, we routinely obtained a specimen of terminal rectum or fistula trimmed (1–3 cm) during PSARP and sent it to pathologists. In our hospital, the procedure of pathologists searching for ganglia was similar to that reported by Midrio et al. (16). The material was extensively examined by two pathologists using a two-step procedure. The first set of twelve 4-μm-thick sections stained with hematoxylin and eosin (H&E) and a final 6-μm-thick section for Calretinin immunohistochemistry was obtained after assessing sample adequacy. If ganglia are found, no further analysis is required. If no ganglia are found, all sections are examined. According to the results of the pathological examination, we divided the patients into two groups: Group 1—ganglia absence and Group 2—ganglia presence.

Spinal MRI (axial and sagittal views) and x-ray (anterior–posterior and lateral views) examinations were performed for all patients to screen spinal cord and vertebral/sacral anomalies before PSARP, and images were evaluated and reported by a pediatric neuroradiologist. Spinal cord anomalies (SCAs) were assessed according to the criteria described in the study by Esposito et al. (17), and sacral ratios (SRs) were calculated from the anterior–posterior or lateral pelvic x-ray examinations using the classic formula proposed by Torres et al. (18). To ensure accuracy, SR was measured three times by the same radiologist in an unknown case, with the mean as the final data.

### Evaluation of defecation function

**Figure 2:**
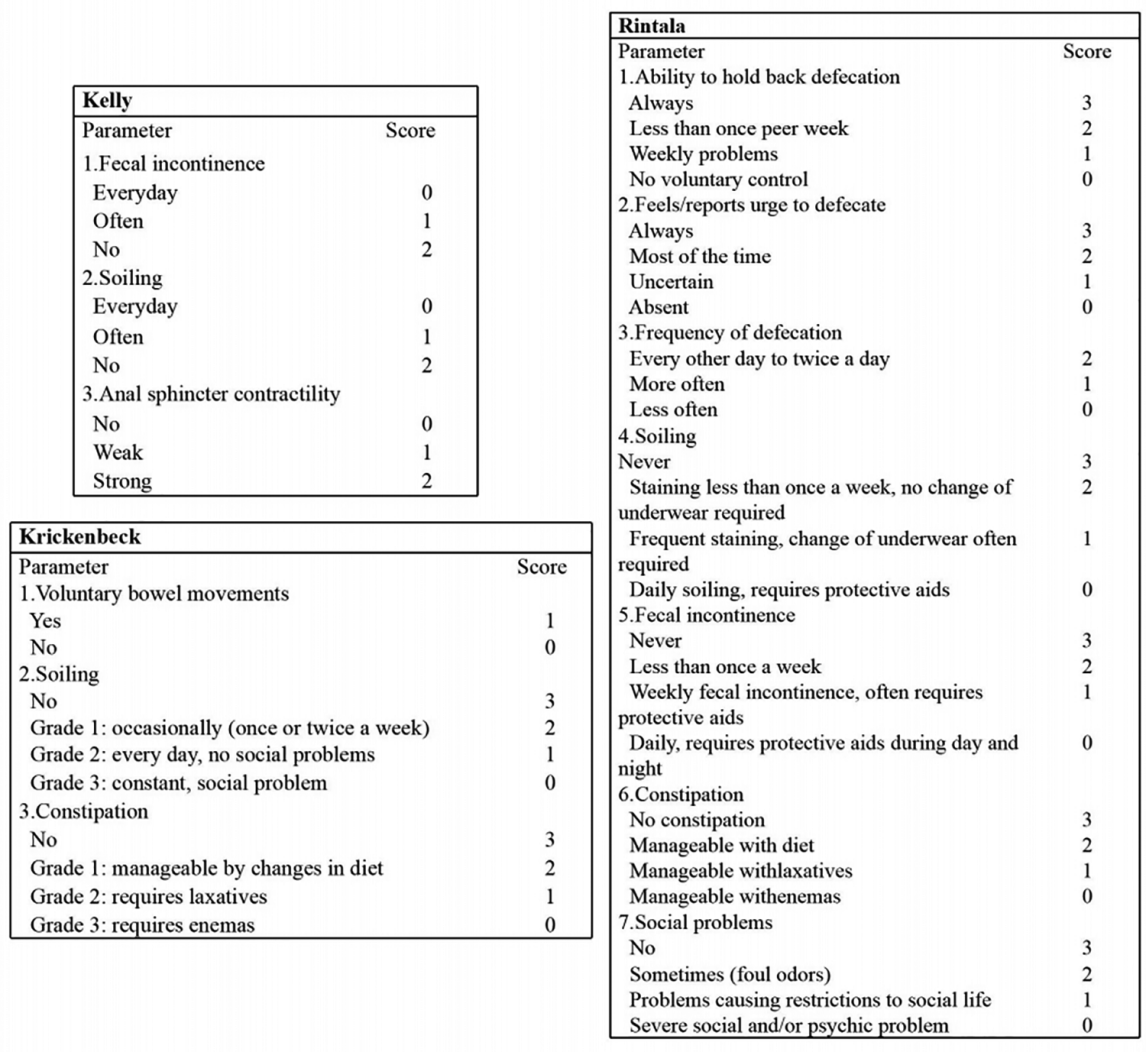
Details of Kelly, Krickenbeck, and Rintala questionnaires to evaluate defecation function.

Defecation function was evaluated using various rating scale questionnaires, including Kelly, Krickenbeck, and Rintala questionnaires, conducted by two experienced surgeons. The reasons for choosing three protocols for evaluation are as follows: the Kelly questionnaire is designed to evaluate the severity of fecal incontinence, while the Krickenbeck questionnaire is for constipation. Furthermore, the Rintala questionnaire is the only one that has been validated on healthy children. The Kelly questionnaire consists of items related to fecal incontinence, soiling, and anal sphincter contractility. Each of these three parameters is scored as 0–2 according to the degree of impairment, with a variable final score ranging from 0 to 6 (Figure 2). According to Kelly's definition, patients with a score ranging from 5 to 6 are considered to have “good” continence, from 3 to 4 “fair,” and from 0 to 2 “poor” (6). The Krickenbeck questionnaire, as described in Figure 2, includes three parameters: voluntary bowel movements, soiling, and constipation. With this questionnaire, patients had a variable final score ranging from 1 to 7, and lower scores indicated worse defecation ability (6). The Rintala questionnaire, for children older than 3 years, consists of seven parameters: the ability to hold back defecation, feeling/reporting an urge to defecate, frequency of defecation, soiling, fecal incontinence, constipation, and social problems (Figure 2) (6). Patients were classified into four categories according to their scores, as follows: normal (18–20), good (12–17), fair (7–11), and poor (0–6) (19, 20).

### Statistical analysis

Excel software was used for data entry, and statistical analysis was performed using SPSS 22.0 software. Categorical data were expressed by *n* (%), and Fisher's exact test and the chi-squared test were used for comparison, as appropriate. Continuous data were expressed as the mean ± standard deviation (SD) and compared using Student's *t*-test. The area under the curve (AUC) of the receiver operating characteristic curve (ROC) was used to quantify the predictive value of no ganglia on postoperative constipation with sensitivity/specificity. A *P*-value less than 0.05 was considered statistically significant.

## Results

### General data

The total number of patients during the time frame of this study was 72; of these, 62 were eligible and 10 were excluded, as detailed in Figure 1. Of the 62 patients, 38 cases (61.3%) had imperforate anus with RB fistulas and 24 cases (38.7%) had imperforate anus with RP fistulas. Most of the cases (62.9%) lived in rural areas. By analyzing the imaging data, spinal anomalies (vertebral and/or sacral anomalies) were found in 30.6% (19/62) of the patients and SCAs were found in 56.5% (35/62). The most common SCA was spinal dysraphism (38.7%), followed by tethered spinal cord (TSC, 24.2%) and syrinx (11.3%). According to the results of the pathological examination, 18 cases (29.0%) were included in Group 1 and 44 cases (71.0%) were classified as Group 2.

### Correlations of ganglia grouping with baseline information

The baseline information of these patients is shown in Table 1. The patients in Groups 1 and 2 were comparable in terms of demographic data (birth weight, gestational age, type of ARM, residence), excision length, the various ages (Ages 1–4) receiving interventions or evaluations, and imaging study results (spinal anomalies, SCAs, and anterior–posterior and lateral SR views) (all *P*’s > 0.05).

**Table 1:** Correlations of ganglia grouping with demographic data of patients with RB/RP fistulas.

Parameters	Group 1, *n* = 18	Group 2, *n* = 44	*P*-value
Birth weight (g)	3016.6 ± 359.0	2996.0 ± 415.6	0.855
Gestational age (weeks)	38.6 ± 1.6	38.4 ± 1.8	0.444
Type of ARM (*n*, %)			0.082
RB fistula	8 (44.4)	30 (68.2)	
RP fistula	10 (55.6)	14 (31.8)	
Residence (*n*, %)			0.852
Rural	11 (61.1)	28 (63.6)	
Urban	7 (38.9)	16 (36.4)	
Excision length (cm)	1.89 ± 0.47	2.03 ± 0.51	0.298
Age 1 (days)	1.7 ± 0.7	1.7 ± 1.0	0.862
Age 2 (months)	6.4 ± 1.5	6.3 ± 1.4	0.881
Age 3 (months)	9.6 ± 1.6	9.3 ± 1.2	0.439
Age 4 (months)	55.9 ± 9.1	60.2 ± 10.6	0.137
Spinal anomalies (*n*, %)	5 (27.8)	14 (31.8)	0.754
Sacral anomalies (*n*, %)	4 (22.2)	7 (15.9)	0.555
Vertebral anomalies (*n*, %)	1 (5.6)	8 (18.2)	0.200
SCA (*n*, %)	10 (55.6)	25 (56.8)	0.927
Arachnoid cyst (*n*, %)*	1 (5.6)	5 (11.4)	0.662
Conus anomalies (*n*, %)*	1 (5.6)	3 (6.8)	1.000
Spinal dysraphism (*n*, %)	6 (25.0)	18 (31.6)	0.578
Syrinx (*n*, %)*	4 (22.2)	3 (6.8)	0.179
TSC (*n*, %)	4 (22.2)	11 (25.0)	0.817
Anterior–posterior SR	0.65 ± 1.4	0.70 ± 0.10	0.057
Lateral SR	0.71 ± 0.11	0.74 ± 0.09	0.315

* Fisher's exact test.

### Comparison of defecation function

**Figure 3:**
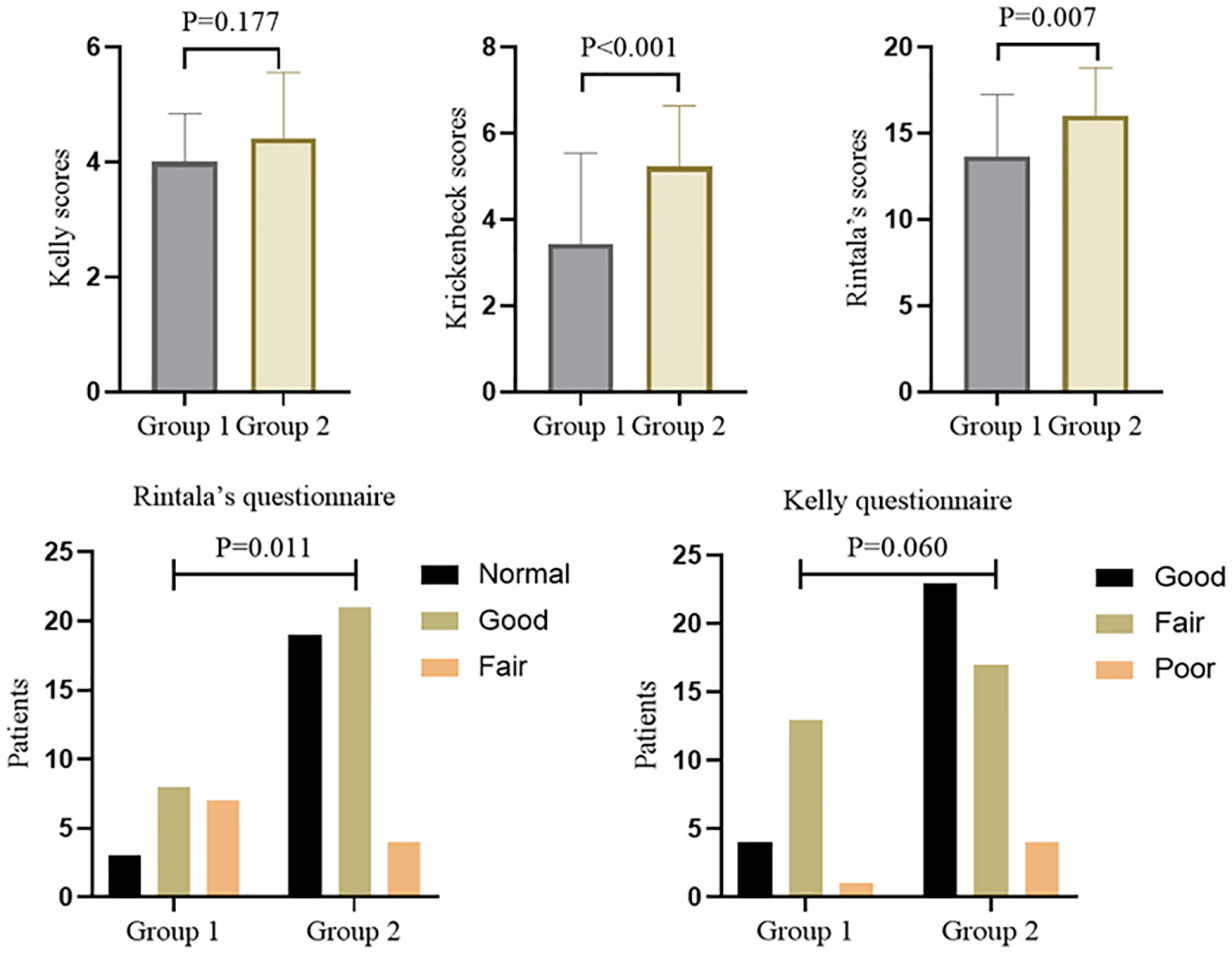
Comparison of defecation function in different groups.

To comprehensively evaluate whether ganglia absence or presence in the terminal rectum/fistula of complex ARM was related to postoperative defecation function, we used three rating scale questionnaires with different focuses. The total scores of the Kelly, Krickenbeck, and Rintala questionnaires in overall patients were 4.3 ± 1.1, 4.7 ± 1.8, and 15.3 ± 3.2, respectively. For the results of the defecation function (Figure 3), there were no significant differences in Kelly scores (4.0 ± 0.8 vs. 4.4 ± 1.1) between Groups 1 and 2 (*P* = 0.177), while Krickenbeck (3.7 ± 1.8 vs. 5.2 ± 1.4) and Rintala (13.7 ± 3.6 vs. 16.0 ± 2.7) scores in Group 1 were significantly lower than those in Group 2 (both *P*’s  < 0.05), consistent with the comparison of categorical data (Group 1 vs. Group 2, Kelly questionnaire: good 22.2%, fair 72.2%, poor 5.6% vs. good 52.3%, fair 38.6%, poor 9.1%; Rintala questionnaire: normal 16.7%, good 44.4%, fair 38.9% vs. normal 43.2%, good 47.7%, fair 9.1%).

### Ganglia absence related to postoperative constipation

**Figure 4:**
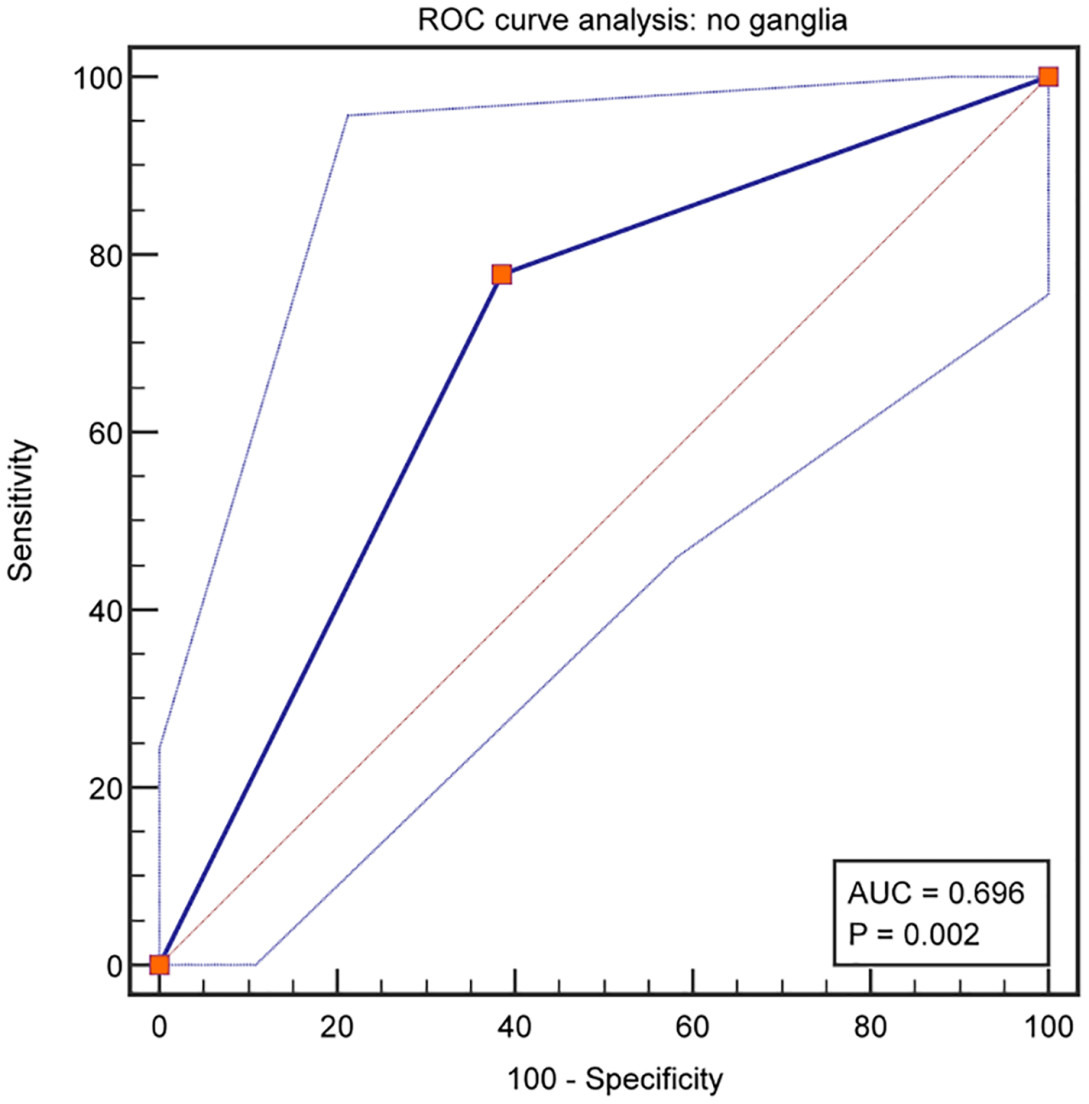
Predictive assessment of ganglia absence for patients with postoperative constipation.

**Figure 5:**
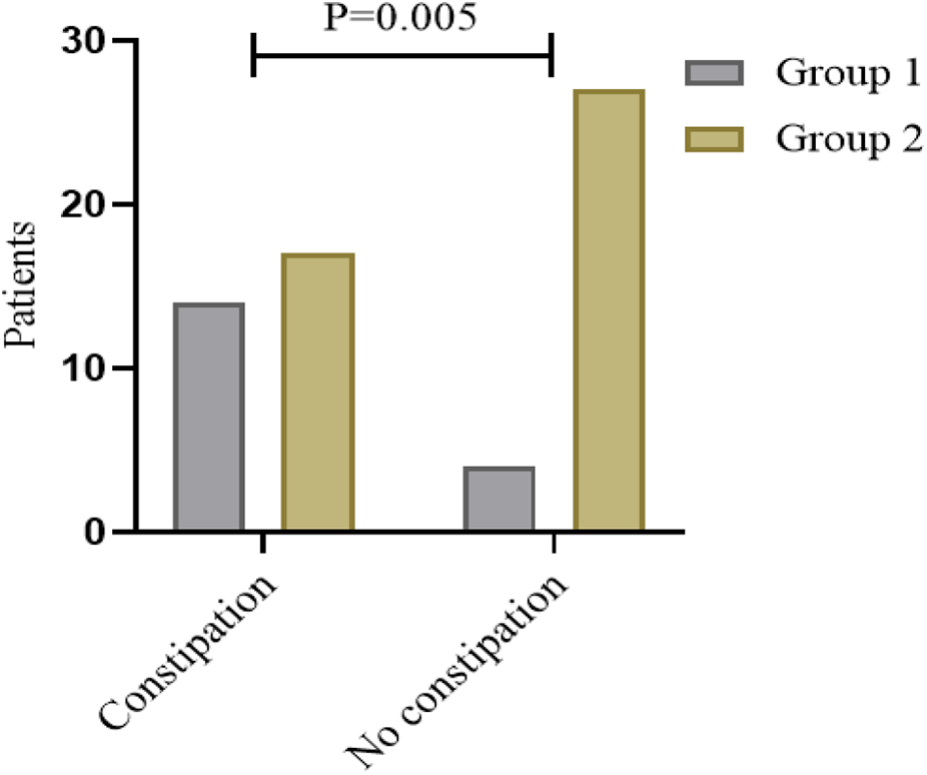
Number of patients with postoperative constipation in different subgroups.

From the analysis of defecation function, we found that the absence of ganglia in the terminal rectum/fistula was not related to Kelly scores (evaluation of fecal incontinence) but Krickenbeck (evaluation of constipation) and Rintala (comprehensive evaluation of defecation function) scores. This suggests that ganglia absence in the terminal rectum/fistula may be related to postoperative constipation. To evaluate whether ganglia absence could accurately predict the patients who had postoperative constipation, we performed a detailed analysis of the Krickenbeck scores (three parameters: voluntary bowel movements, soiling, and constipation), and ROC was used to quantify the predictive value. We found constipation in 31 of the 62 patients (50.0%): in 77.5% of patients in Group 1 and 38.6% of patients in Group 2 (*P* = 0.002, Table 2). The distribution of constipation in Group 1 was significantly higher than that in Group 2. For constipation, the AUC of no ganglia was 0.696 (95% confidence interval: 0.566–0.806, *P* = 0.002, Figure 4), with 77.8% sensitivity and 61.4% specificity. Compared with the presence of ganglia, no ganglia had a 4.559 times higher chance of postoperative constipation (95% confidence interval: 1.567–19.717, Figure 5).

**Table 2:** Detailed analysis of Krickenbeck scores between the two groups.

Parameters	Group 1, *n* = 18	Group 2, *n* = 44	*P*-value
Voluntary bowel movements (*n*, %)			0.084
Yes	11 (61.1)	36 (81.8)	
No	7 (38.9)	8 (18.2)	
Soiling (*n*, %)			0.139
No	2 (11.1)	15 (34.1)	
Grade 1	10 (55.6)	18 (40.9)	
Grade 2	3 (16.7)	9 (20.5)	
Grade 3	3 (16.7)	2 (4.5)	
Constipation (*n*, %)			0.002
No	4 (22.2)	27 (61.4)	
Grade 1	5 (27.8)	10 (22.7)	
Grade 2	5 (27.8)	7 (15.9)	
Grade 3	4 (22.2)	0 (0.0)	

## Discussion

In this study, we conducted a single-center study to evaluate the relationship between ganglionic distribution in the terminal rectum/fistula of complex ARMs and postoperative defecation function. Anal reconstruction was performed by the permanent surgical team in a series of cases of male imperforate anus with RB/RP fistulas matched for demographics and incidence of spinal (cord) anomalies to minimize bias from other variables. The major findings of the study are as follows: (1) ganglia absence was found in the terminal rectum/fistula in 29.0% (18/62) of the patients; (2) ganglia absence was associated with postoperative FDD, mainly constipation; (3) 50% (31/62) of the patients faced varying degrees of constipation, and ganglia absence had limited predictive value for postoperative constipation (AUC: 0.696).

Currently, several problems are concerning pediatric surgeons: (1) should the search for ganglia in the terminal rectum/fistula of ARMs be abandoned after surgery; (2) whether or not we should preserve the terminal rectum/fistula in the reconstruction of ARMs; (3) whether an intraoperative frozen section is necessary in the search for ganglia to determine the excision extension. Midrio et al. suggested that the practice of searching for ganglia after surgery is not meant for detecting associated colorectal diseases and should be abandoned to save medical resources (16). Histomorphological and immunohistochemical reports of the terminal rectum/fistula vary in different studies, resulting in the controversy of preserving or excising the distal segment (10–12, 15). Abnormalities of the innervation pattern in the terminal rectum/fistula are commonly seen in specimens taken during reconstruction. For people without anorectal diseases, the ganglia are normally absent 0.6–2 cm above the dentate line, and this area is called the anal transition zone (21). It has the following physiological functions: controlling the frequency of defecation and continence and the ability to defer defecation or discriminate flatus from feces (22). It has been reported that the terminal rectum/fistula of ARMs is usually located in this area, and ganglia may be physiologically absent (15, 16). However, the question is raised of whether there really is a so-called anal transition zone when the anus is atresia in patients with complex ARMs.

In view of this, we have carefully analyzed these reports and concluded the following reasons. (1) Bias due to differences in the inclusion of study subjects, especially low ARMs, and same or similar pathological types (Krickenbeck classification) may be more comparable. (2) Inconsistent specimen acquisition times and delays in surgery times may cause secondary dilatation of the terminal rectum/fistula, affecting pathological results and postoperative defecation function. (3) Surgical proficiency and specimen's length obtained also influenced the analysis results. Therefore, we conducted this study to optimize the inclusion of subjects and unified surgical procedures as much as possible to make the results more reliable.

Furthermore, we used three common rating scale questionnaires (Kelly, Krickenbeck, and Rintala questionnaires) with different focuses to evaluate the defecation function of patients comprehensively. The Kelly questionnaire is designed to evaluate the severity of fecal incontinence, while the Krickenbeck questionnaire is for constipation. Moreover, the Rintala questionnaire is the only one that has been validated on healthy children. From the results, we found that patients with ganglia presence in the terminal rectum/fistula had higher Krickenbeck and Rintala scores (both *P*'s < 0.05), and patients with ganglia absence had postoperative constipation (*P* < 0.05). In addition, the AUC of no ganglia for predicting constipation was 0.696, with 77.8% sensitivity and 61.4% specificity. We can preliminarily conclude that the factor of ganglia absence had limited predictive value for postoperative constipation, indicating that denervation of the rectum may not be the only cause in the pathogenesis of constipation after PSARP. In future research, other indicators combined with this factor (ganglia absence) may improve the prediction efficiency.

The development of the enteric nervous system (ENS) in the terminal rectum/fistula of ARMs is a research hotspot of this disease, whether it is from the pathogenesis or postoperative defecation function (23, 24). The ENS has varying degrees of abnormality in complex ARMs (including the absence of ganglia, resulting in bowel motility dysfunction and the clinical manifestation of constipation), but the specific molecular mechanisms remain unclear (25–28). Embryological studies have initially elucidated the mechanism of ENS genesis: it originates from vagal and sacral neural crest cells and is constructed by intermuscular and submucosal ganglia (29). The migration, colonization, and differentiation of enteric neural crest-derived cells (ENCDCs) from head to tail are important processes for the development of ENS (30). ENCDCs initially differentiate into neurons or neuroglia, which aggregate between the circumferential and longitudinal muscular layers to form intermuscular ganglia; while the other part continues to migrate radially from the intermuscular layer to the submucosal layer to develop into submucosal ganglia. Because the rectum is in the terminal position of the digestive tract, abnormalities in any link throughout the process of ENS development may lead to the absence of terminal rectal ganglia.

Our study may provide two useful references for the clinical treatment of male imperforate anus with RB/RP fistulas. First, searching for ganglia in the terminal rectum/fistula, followed by anoplasty to determine the region of excision, may be a viable option, as this procedure seems to help reduce postoperative constipation. Second, pediatric surgeons need to be aware of postoperative defecation function in order to provide proper solutions and parental counseling. If the postoperative pathological findings show the absence of ganglia in the terminal rectum/fistula, surgeons need to adjust parental expectations and encourage them to plan for a bowel management program at the age of toilet training. Thus, searching for ganglia in the terminal rectum/fistula after anoplasty is also important. Further studies comparing the functional outcomes of terminal rectum/fistula preservation and resection in other complex types of ARMs may be required, such as rectobladderneck fistulas and cloacal anomalies.

## Limitations

There are several limitations in this retrospective study. First, it is a single-center retrospective study, and there is a potential bias in data collection and study population selection. The cohort here does not necessarily represent the entire population of complex ARMs, and a prospective multicenter study based on consistent standards is important to gain more reliable results. Also, this evaluation, provided by the patient's guardian, only captures a snapshot of defecation function outcomes at one point in time, as opposed to changes over a longer period in childhood. Finally, the sample size is relatively small. Statistically, there is no significant difference between Groups 1 and 2 in the categorical data of the Kelly questionnaire; however, it shows a boundary value (*P* = 0.060), and clinical conclusions are difficult to draw completely due to the small number.

## Conclusion

The problem of defecation function after the reconstruction of ARM is still a challenge for pediatric surgeons, especially complex types of ARMs (6). By optimizing the design, this study shows that ganglia absence in the terminal rectum/fistula of male imperforate anus with RB/RP fistulas is associated with constipation after anoplasty, but it has limited predictive value for postoperative constipation. The results of this study suggest the need to search for ganglia in the terminal rectum/fistula, both intraoperatively and postoperatively.

## Data Availability

The original contributions presented in the study are included in the article/Supplementary Material, further inquiries can be directed to the corresponding author.
